# *Aspergillus* identification through bronchoscope in intensive care unit – a retrospective, databased cohort study

**DOI:** 10.3389/fcimb.2024.1471298

**Published:** 2025-01-13

**Authors:** Hsin-I Cheng, Chun-Yu Lin, Horng-Chyuan Lin, Shu-Min Lin, Meng-Heng Hsieh, Yueh-Fu Fang, Po-Jui Chang, Wei-Syun Hung, Ko Cheng, Chung−Chi Huang

**Affiliations:** ^1^ Department of Thoracic Medicine, Chang Gung Memorial Hospital, Taoyuan, Taiwan; ^2^ School of Medicine, Chang Gung University, Taoyuan, Taiwan; ^3^ Department of Respiratory Therapy, Chang Gung Memorial Hospital, Taoyuan, Taiwan; ^4^ School of Medicine, National Tsing Hua University, Hsin-Chu, Taiwan; ^5^ Center for Big Data Analytics and Statistics, Chang Gung Memorial Hospital, Taoyuan, Taiwan

**Keywords:** Aspergillus, invasive pulmonary aspergillosis (IPA), galactomannan (GM), intensive care unit (ICU), broncho alveolar lavage (BAL)

## Abstract

**Introduction:**

Invasive pulmonary aspergillosis (IPA) increases the risk of mortality of critically ill patients. Diagnostic criteria specifically targeting patients in intensive care units(ICUs) have been developed to improve diagnostic sensitivity. This study investigated health outcomes among patients in ICUs with Aspergillus isolates identified using bronchoscopy.

**Methods:**

This retrospective cohort study obtained data from the Chang Gung Research Database of Chang Gung Memorial Hospital. Patients admitted to the ICU between January 2017 and December 2022 who received bronchoalveolar lavage were enrolled. Patients with a fungus culture yielding Aspergillus spp. isolates or who had an Aspergillus galactomannan antigen index value of >1.0 were categorized into the Aspergillus-positive group.

**Results:**

A total of 2372 patients were enrolled, and 146 patients (6.16%) tested positive for Aspergillus. Of the patients who tested positive for Aspergillus, 37.67% had a positive culture result, and 77.4% had a positive galactomannan antigen result. Patients with Aspergillus isolates were more likely to have a recent influenza infection, concurrent bacterial sepsis, and a cavitation and to die in hospital (in-hospital mortality rate 58.9% vs. 48.57%, P = 0.016).

**Discussion:**

Identifying Aspergillus through bronchoscopy in the ICU is associated with higher mortality rates than in patients who test negative for Aspergillus. Galactomannan antigen from bronchoalveolar lavage may provide higher diagnostic sensitivity.

## Introduction

Invasive pulmonary aspergillosis (IPA) frequently occurs as an opportunistic infection in intensive care units (ICUs) and has been associated with increased risks of morbidity and mortality, particularly in immunocompromised individuals. Previous research has shown that ICU patients diagnosed with IPA face high mortality rates (Meersseman et al., 2004; Ku et al., 2017; Lin et al., 2017), with IPA-associated tracheobronchitis mortality reached 93.5% (Lin et al., 2017). IPA even increases the risk of death among nonneutropenic patients (Ku et al., 2017).

Diagnostic criteria for IPA were established in 2002 by the European Organization for Research and Treatment of Cancer Invasive Fungal Infections Cooperative Group and the National Institute of Allergy and Infectious Diseases Mycoses Study Group (EORTC/MSG). These criteria were then updated in 2008 and 2020 to enhance their utility in research and in clinical settings (Ascioglu et al., 2002; De Pauw et al., 2008; Donnelly et al., 2020). These criteria predominantly target immunocompromised populations.

*Aspergillus* spp. can cause invasive diseases in diverse patient groups (Coulon et al., 2020; Nyga et al., 2020; Kuo et al., 2022; Hatzl et al., 2024). Worldwide, an estimated 519,000 patients in ICUs may be affected by IPA (Denning, 2024). According to one study, mortality among patients in ICUs with IPA who did not receive treatment exceeded 95% (Denning, 2024). Diagnosing IPA is particularly challenging in clinical settings. Standard diagnostic definitions, developed primarily for patients with cancer or patients who have undergone hematopoietic stem cell transplant, may not apply to critically ill patients, who often lack the host factors specified in EORTC criteria. Obtaining histological diagnoses for critically ill patients is also difficult. Diagnostic criteria that can be effectively applied to this patient group are warranted.

Several algorithms—including AspICU, BM-AspICU, and Modified AspICU—have been developed for use as IPA diagnostic tools in ICUs (Blot et al., 2012; Schauwvlieghe et al., 2018; Hamam et al., 2021). These algorithms can be used to determine the risk of IPA in patients who have had influenza, patients with neutropenia, patients who have received systemic corticosteroid treatment, and patients who have undergone stem cell transplant; however, whether they can be used with other patient groups is uncertain. Our objective was to compare the prognosis of patients with *Aspergillus* isolates identified using bronchoscopy against other patient groups.

## Method

### Data source

Patients in ICUs between 2017 and 2022 were identified from the Chang Gung Research Database (CGRD), which belongs to the Chang Gung Medical Foundation. This foundation is the largest hospital system in Taiwan, comprising three medical centers (in Linkou, Taipei, and Kaohsiung) and four regional hospitals (in Taoyuan, Keelung, Chiayi, and Yunlin) located across Taiwan. The CGRD contains patients’ demographic data, inpatient and outpatient records, diagnostic codes, medication records, microbiological data, imaging study reports, and functional examination data (Lin et al., 2022). Disease diagnoses are coded in the database by using the *International Classification of Diseases, Tenth Revision*. This study received approval from the Institutional Review Board of the Chang Gung Memorial Foundation (IRB No. 202301837B0). Due to the retrospective nature of the study, the requirement for informed consent was waived.

### Study design

Patients admitted to an ICU between January 2017 and December 2022 who required mechanical ventilation and who underwent bronchoscopy and bronchoalveolar lavage (BAL) were enrolled and divided into two groups. Patients with a fungus culture from BAL fluid yielding *Aspergillus* spp. isolates or who had an *Aspergillus* galactomannan antigen index value of >1.0 were categorized into the *Aspergillus*-positive group. The remaining patients were categorized into the *Aspergillus*-negative group. Patients were excluded if they were aged <18 years, had human immunodeficiency virus, or did not have data on Acute Physiology and Chronic Health Evaluation II (APACHE II) scores obtained upon their ICU admission.

### Covariates and outcomes

The following covariates were analyzed: age, sex, chronic comorbidities (d iabetes mellitus, heart failure, liver cirrhosis, chronic renal insufficiency, cancer, hematological malignancy, chronic obstructive pulmonary disease, prior tuberculosis infection, autoimmune disease, and organ transplant), and the following patient conditions: APACHE II score, acute kidney injury requiring renal replacement therapy, length of ICU stay, length of mechanical ventilation use, length of hospital stay, neutropenia prior to bronchoscopy (defined as absolute neutrophil count ≤500 cells/mm^3^), concurrent bacterial sepsis (defined as any positive bacterial culture of blood, BAL fluid, or sputum within the 1 week before and after entering or leaving the ICU), imaging reports from radiologists, serum and BAL fluid galactomannan antigen indices, fungus culture results, and antifungal treatment status. Patients were defined as having a comorbidity if they had at least two outpatient diagnoses or one inpatient diagnosis for that comorbidity prior to the index date. The patterns of image reports were defined by reports from radiologists. Antifungal treatment status was defined as adequate or inadequate on the basis of whether the patient was administered any dose of voriconazole, posaconazole, isavuconazole, caspofungin, amphotericin B, or liposomal amphotericin B.

### Statistical analysis

Results are presented as means with standard deviations or as numbers and percentages. Student’s t test for independent samples was used to compare continuous variables that followed a normal distribution. Pearson’s chi-square test or Fisher’s exact test were used to compare categorical variables. The Mann–Whitney U test was employed to compare continuous variables that did not follow a normal distribution, which occurred in several subgroup analyses involving only a few patients. Statistical significance was set at a two-sided P value of < 0.05.

## Results

**Figure 1:**
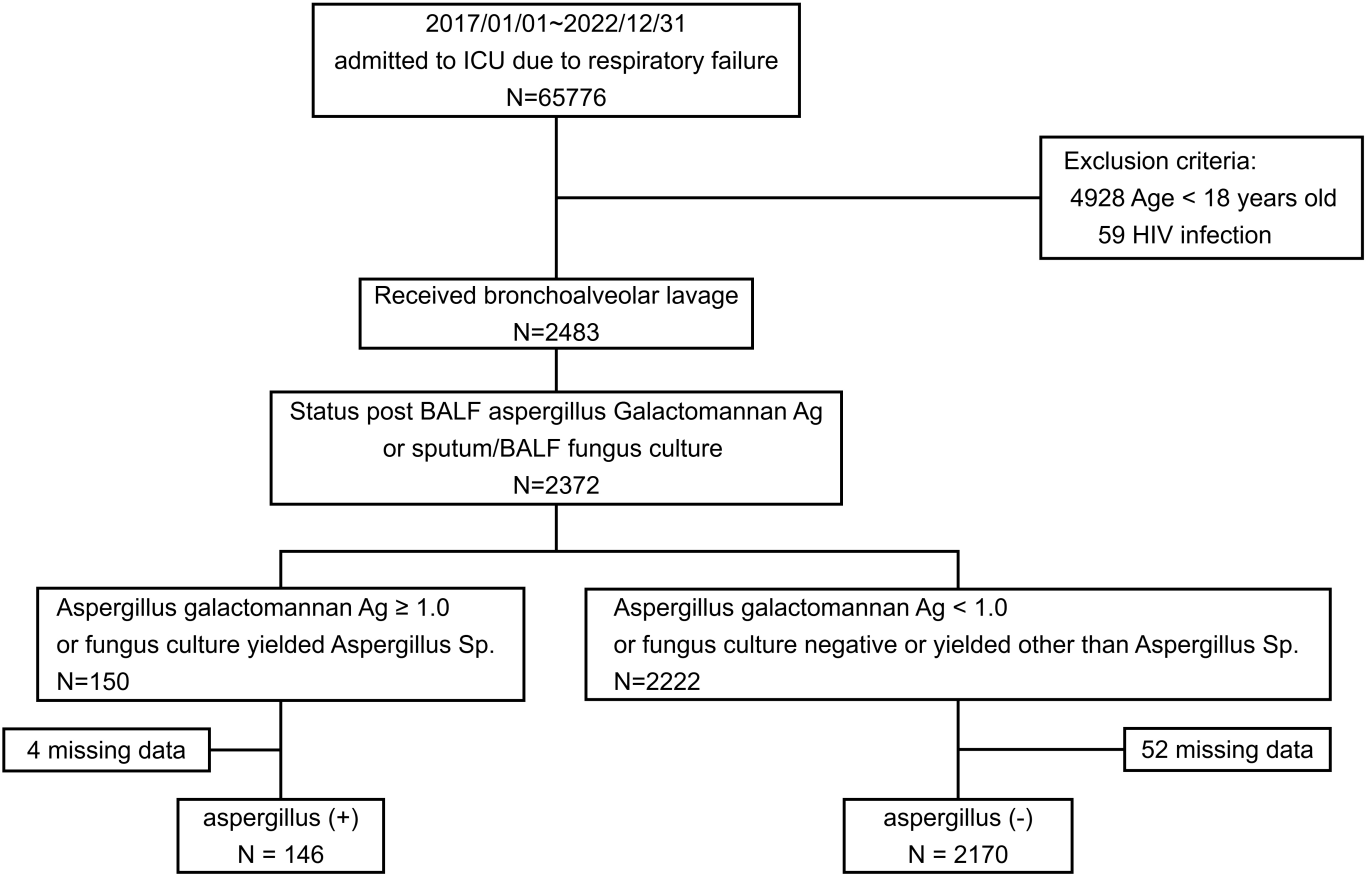
Study flowchart.

Between January 1, 2017, and December 31, 2022, BAL procedures were conducted on 2483 patients. Results for either the *Aspergillus* galactomannan antigen index or sputum BAL fluid fungus culture were available for 2372 of these patients. In total, 146 patients tested positive for *Aspergillus*, and 2170 tested negative for *Aspergillus* (**Figure 1**). Overall, 6.16% of the patients with positive galactomannan antigen or fungus culture results tested positive for *Aspergillus*.

### Baseline characteristics

In the *Aspergillus*-positive group, 113 patients (77.4%) had a positive result for the *Aspergillus* galactomannan antigen index in BAL fluid. Additionally, 55 patients (54.79%) had a positive result from a fungal culture, while 22 patients (15.1%) tested positive in both the fungal culture and the galactomannan antigen index. The mean age of the patients in this study was 66.4 years, and approximately one-third (707, 30.53%) of the patients were women (**Table 1**). The most common chronic comorbidity was cancer (915, 39.51%). No differences in the prevalence of chronic comorbidities were discovered between the *Aspergillus*-positive and *Aspergillus*-negative groups except for hematological malignancy (9.85% in the *Aspergillus*-positive group and 5.58% in the *Aspergillus*-negative group, P = 0.045). The *Aspergillus*-positive group was more likely to experience postinfluenza infection than was the *Aspergillus*-negative group (6.85% vs. 2.21%, P = 0.005). No difference in the rate of post-COVID-19 infection was found between the groups.

**Table 1:** The characteristics of patients with and without *Aspergillus* identification.

Baseline Characteristics	All N=2316	*Aspergillus* (+) N=146	*Aspergillus* (-) N=2170	P value
Age, yr (mean±S.D.)	66.4 ± 14.5	67.7 ± 13.5	66.3 ± 14.6	0.2512
Female sex, no. (%)	707 (30.53)	46 (31.51)	661 (30.46)	0.7905
Chronic co-existing condition, no. (%)				
Diabetes mellitus	36 (1.55)	0 (0.00)	36 (1.66)	0.1663
Heart failure	124 (5.35)	5 (3.42)	119 (5.48)	0.2847
Liver cirrhosis	89 (3.84)	4 (2.74)	85 (3.92)	0.6557
Chronic renal insufficiency	178 (7.69)	12 (8.22)	166 (7.65)	0.8026
Cancer	915 (39.51)	64 (43.84)	851 (39.22)	0.2691
Hematological malignancy	135 (5.83)	14 (9.59)	121 (5.58)	0.0451
Structure lung disease	252 (10.88)	18 (12.33)	234 (10.78)	0.5616
Bronchiectasis	39 (1.68)	4 (2.74)	35 (1.61)	0.3058
Chronic obstructive pulmonary disease	227 (9.80)	16 (10.96)	211 (9.72)	0.6270
Post TB infection	57 (2.46)	4 (2.74)	53 (2.44)	0.7802
Autoimmune disease	116 (5.01)	8 (5.48)	108 (4.98)	0.7876
Post organ transplant	54 (2.33)	3 (2.05)	51 (2.35)	1.0000
COVID-19 coinfection	41 (1.77)	3 (2.05)	38 (1.75)	0.7414
Influenza coinfection	58 (2.50)	10 (6.85)	48 (2.21)	0.0005

Values are listed as median [IQR] or number (%).

TB, Tuberculosis.

COVID-19, Coronavirus disease 2019.

### Clinical outcomes

**Figure 2:**
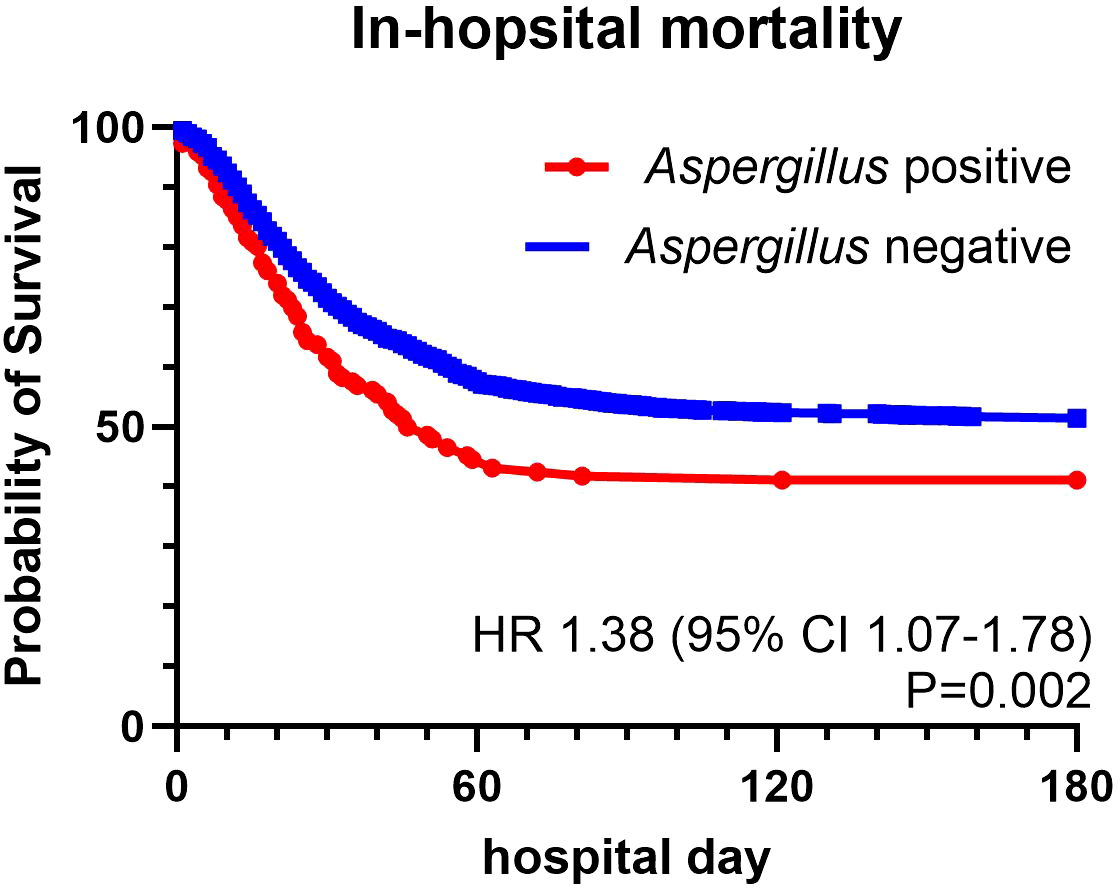
Survival curves for patients with or without aspergillus identification. hazard ratio: 1.38; 95% Confidence Interval: 1.07-1.78, Log-rank Test P =0.002.

The overall mortality rate was 49.22% (**Table 2**). The mortality rate was significantly higher in the *Aspergillus*-positive group than in the *Aspergillus*-negative group (58.9% vs. 48.57%, P = 0.016). No differences in the incidence of acute kidney injury requiring renal replacement therapy, length of ICU stay, length of mechanical ventilation use, length of hospital stay, ICU mortality rate, or ventilator dependency were found. The *Aspergillus*-positive group had a higher risk of concurrent bacterial sepsis than did the *Aspergillus*-negative group (40.41% vs. 31.34%, P = 0.023). Computed tomography revealed a higher proportion of cavitation in the *Aspergillus*-positive group than in the *Aspergillus*-negative group (7.53% vs. 3.59%, P = 0.017). The treatment outcomes for the *Aspergillus*-positive group are listed in **Table 3**. Although patients who survived were more likely than were those who did not survive to have received effective antifungal treatment, the disparity was nonsignificant. Kaplan–Meier survival analysis was used to compare the mortality rates of the *Aspergillus*-positive and *Aspergillus*-negative groups (**Figure 2**); the in-hospital mortality rate was found to be significantly higher in the *Aspergillus*-positive group (hazard ratio: 1.38, reference: *Aspergillus*-negative group; 95% confidence interval: 1.07–1.78, log-rank test P = 0.002). The *Aspergillus* spp. identified in fungus cultures are listed in **Table 4**.

**Table 2:** The characteristics of patient with and without *Aspergillus* identification in ICU.

Variable	All N=2316	*Aspergillus* (+) N=146	*Aspergillus* (-) N=2170	P value
APACHE II score on ICU admission	20 [15-25]	20 [15-26]	20 [15-25]	0.5639
AKI requiring RRT	296 (12.78)	13 (8.90)	283 (13.04)	0.5941
Length of ICU stay	23 [11-35]	20 [9-32]	23 [11-35]	0.2146
Length of MV use	16 [9-27]	17 [10-31]	16 [9-27]	0.5168
Length of hospital stay	23 [10-37]	20 [9-33]	23 [10-38]	0.3172
Neutropenia prior to bronchoscopy	44 (1.90)	1 (0.68)	43 (1.98)	0.5226
Concurrent bacterial sepsis	739 (31.91)	59 (40.41)	680 (31.34)	0.0228
Ventilator dependent	186 (8.03)	13 (8.90)	173 (7.97)	0.6884
In ICU mortality	501 (21.63)	31 (21.23)	470 (21.66)	0.9037
In hospital mortality	1140 (49.22)	86 (58.90)	1054 (48.57)	0.0156
Radiography				
CT positive	1399 (60.41)	88 (60.27)	1311 (60.41)	0.9731
Nodular/mass	891 (38.47)	58 (39.73)	833 (38.39)	0.7475
Consolidation	988 (42.66)	64 (43.84)	924 (42.58)	0.7666
GGOs	697 (30.09)	34 (23.29)	663 (30.55)	0.0639
Cavitation	89 (3.84)	11 (7.53)	78 (3.59)	0.0165
CT missing	826 (35.66)	55 (37.67)	771 (35.53)	–
Diagnosis				
BALF Aspergillus Galactomannan Ag	113 (4.88)	113 (77.40)	0 (0.00)	–
Positive Aspergillus culture	55 (2.37)	55 (37.67)	0 (0.00)	–
Positive in both culture and Ag	22 (0.95)	22 (15.07)	0 (0.00)	–

Values are listed as median [IQR] or number (%).

ICU, intensive care unit.

APACHE, Acute Physiology and Chronic Health Evaluation.

AKI, Acute kidney injury.

RRT, renal replacement therapy.

MV, mechanical ventilation.

CT, computed tomography.

GGO, ground-glass opacity.

BALF, Bronchoalveolar lavage fluid.

Ag, antigen.

**Table 3:** Compared all-cause mortality for drug selection.

Variable	Dead(n=86)	Alive(n=60)	P value
No/inadequate antifungal treatment	57 (66.28)	32 (53.33)	0.1147
Adequate antifungal treatment	29 (33.72)	28 (46.67)	0.1147
Voriconazole (PO/IV)	27 (31.40)	26 (43.33)	0.1400
Posaconazole (PO/IV)	3 (3.49)	1 (1.67)	0.6438
Caspofungin (PO/IV)	4 (4.65)	2 (3.33)	1.0000
Amphotericin B	5 (5.81)	2 (3.33)	0.7003
Liposomal Amphotericin B	4 (4.65)	0 (0.00)	0.1438
Combination therapy	11 (12.79)	2 (3.33)	0.0740

Values are listed as number (%).

PO, per os, Oral administration.

IV, Intravenous administration.

**Table 4:** *Aspergillus* species in fungus culture.

Species	number
*Aspergillus* spp.	55
*Aspergillus fumigatus*	23
*Aspergillus flavus*	15
*Aspergillus niger*	3
*Aspergillus terrus*	6
Other Aspergillus^*^	3
Undifferentiated *Aspergillus* species^#^	5

*Other Aspergillus defined as an Aspergillus species that is distinct from Aspergillus fumigatus, Aspergillus flavus, Aspergillus niger, or Aspergillus terreus.

# Undifferentiated *Aspergillus* species defined as the exact species of *Aspergillus* could not be determined.

## Discussion

The present study represents a pioneering approach to examining the occurrence of *Aspergillus* spp. in BAL samples collected from ICU patients. In total, 6.16% of our study cohort tested positive for *Aspergillus*. Patients with postinfluenza infection were more likely to test positive for *Aspergillus*. In-hospital mortality was higher among the patients who tested positive for *Aspergillus* than among those who tested negative; however, in-ICU mortality was not correlated with *Aspergillus* status.

Other studies have reported IPA prevalence ranging from 5% to 7% in nonimmunocompromised patients in ICUs (Baddley et al., 2013; Tudesq et al., 2019), similar to the rate obtained in the present study. IPA was shown to occur in 12.5% of patients with acute respiratory distress syndrome, as determined by autopsy (Tudesq et al., 2019). Also, the positive and negative predictive values of AspICU were only 61% and 92%, respectively. Negative predictive values may be as low as 71% in nonimmunocompromised individuals (Blot et al., 2012). This suggests that the prevalence of IPA among nonimmunocompromised patients in ICUs may be somewhat underestimated.

The mortality rate in the present study was 58.9% and 48.6% in the *Aspergillus*-positive and *Aspergillus*-negative groups, respectively. IPA-associated mortality differs across patient categories. In one study, the in-hospital mortality rate among patients with influenza-associated pulmonary aspergillosis was 49% (Schauwvlieghe et al., 2018). In another study, which involved patients with acute respiratory distress syndrome and IPA, the in-ICU mortality rate was 60% (Contou et al., 2016). A study involving patients with hematological malignancies and IPA and receiving invasive mechanical ventilation reported a 90-day mortality rate of 80.4% (Pardo et al., 2019). A systemic review and meta-analysis performed in 2022 reported an IPA-associated mortality rate of 54% (Shi et al., 2022). An article recently published in *Lancet Infectious Diseases* revealed a large discrepancy in mortality rates among patients with IPA in ICUs between those who did and those who did not receive treatment for IPA (50% vs. 95%) (Denning, 2024). In the present study, the *Aspergillus*-positive group did not fully meet any single diagnostic criteria. Mortality rates significantly differed between patients with and without positive results for *Aspergillus.* Blot et al. developed the AspICU algorithm to calculate a 70% mortality rate in patients with putative IPA (Blot et al., 2012). Schauwvlieghe et al. used the modified-AspICU algorithm to calculate a 49% in-hospital mortality rate among patients with IPA (Schauwvlieghe et al., 2018). Our research revealed an in-hospital mortality rate of 58.9% for patients with positive results for *Aspergillus*. These findings suggest that existing diagnostic algorithms are insufficient when employed in ICU settings.

The optimal method for diagnosing IPA in nonimmunocompromised patients in ICUs remains unclear. Delayed diagnoses of IPA could be linked to higher mortality (Inoue et al., 2022). Postponing antifungal treatment for IPA could lead to higher likelihood of in-hospital mortality and a longer hospital stay (Baddley et al., 2013; Denning, 2024). For each day that antifungal therapy initiation is delayed, the length of hospital stay increases by 1.28 days and costs increase by 3.5% (Baddley et al., 2013). Therefore, implementing advanced diagnostic techniques is essential, particularly in cases involving patients with malignancy or recent viral infections, such as COVID-19 or influenza. The galactomannan antigen index proved to be an excellent diagnostic tool, demonstrating high sensitivity and specificity. A meta-analysis revealed sensitivity of 85% and specificity of 86% when employing a cutoff galactomannan antigen index of 1.0 (Zou et al., 2012). The latest EORTC/MSG guidelines recommend a cutoff index of 1.0 for the result of galactomannan antigen enzyme-linked immunosorbent assay for BAL samples. This cutoff yielded sensitivity between 75% and 86% and specificity between 94% and 95%. Sensitivity and specificity values were consistent regardless of the presence of hematological malignancies, with sensitivity ranging from 85% to 87% and specificity from 91% to 89% (Mercier et al., 2021). The AspICU diagnostic criteria do not incorporate galactomannan antigen index values (Blot et al., 2012). Similarly, the Invasive Fungal Diseases in Adult Patients in Intensive Care Unit (FUNDICU) criteria do not cover all ICU patients with predisposing conditions (Bassetti et al., 2024). The mortality rate in the present study’s *Aspergillus*-positive group was comparable to those in other studies and was significantly higher than that in the *Aspergillus*-negative group. Consequently, we propose that being an ICU patient be specified as a host factor in IPA diagnostic criteria. This would facilitate the timely diagnosis of IPA in critically ill patients and improve their health outcomes.

This study has several limitations. First, because the study was retrospective in nature, selection bias may have been present. This bias was potentially mitigated by including only patients who underwent BAL in the ICU. Second, BAL is potentially unsuitable for patients with high oxygen requirements and for those who are hemodynamically unstable. These conditions arise from IPA but also from other factors, possibly leading to an underestimation of the mortality rate in both groups. Third, we were unable to determine whether antifungal agents had been administered for prophylaxis or treatment purposes, nor could we verify whether patients had completed their prescribed courses of treatment. This uncertainty could have resulted in an underestimation of treatment effectiveness. Furthermore, specific immune traits associated with IPA, such as the length and dosage of glucocorticoid treatment and the period of neutropenia, were not discernible. Consequently, these results should be interpreted with caution. Finally, several novel tools for diagnosing IPA, such as polymerase chain reaction (Rath and Steinmann, 2018; Scharmann et al., 2021) and serum IL-8 testing (Heldt et al., 2018), were unavailable in the present study due to equipment constraints. Prospective, large-scale studies are warranted to validate our results.

## Conclusion

Identifying *Aspergillus* through bronchoscopy in the ICU is linked to increased mortality. Existing diagnostic criteria may not be effective when applied to patients in ICUs. Regular evaluation of galactomannan antigen index values obtained through BAL may provide better diagnostic sensitivity, particularly in patients with a recent influenza infection.

## Data Availability

The data analyzed in this study is subject to the following licenses/restrictions: Applicants who fulfill one of the following requirements are eligible to apply: (1) A full-time attending physician of this hospital or a full-time, part-time attending physician, resident, clinical researcher, administrative, medical and technical, or nursing staff of Chang Gung University. (2) Teachers of Chang Gung University and Chang Gung University of Science and Technology who are at the rank of assistant professor or above are required to collaborate on research projects with attending physicians specializing in related disciplines or fields in the Hospital before submitting an application. Requests to access these datasets should be directed to Center for Big Data Analytics and Statistics, chialing@cgmh.org.tw.

## References

[B1] Ascioglu S. Rex J. H. de Pauw B. Bennett J. E. Bille J. Crokaert F.. (2002). Defining opportunistic invasive fungal infections in immunocompromised patients with cancer and hematopoietic stem cell transplants: an international consensus. Clin. Infect. Dis. 34, 7–14. doi: 10.1086/323335 11731939

[B2] Baddley J. W. Stephens J. M. Ji X. Gao X. Schlamm H.T. Tarallo M. (2013). Aspergillosis in Intensive Care Unit (ICU) patients: epidemiology and economic outcomes. BMC Infect. Dis. 13, 29. doi: 10.1186/1471-2334-13-29 23343366 PMC3562254

[B3] Bassetti M. Giacobbe D. R. Agvald-Ohman C. Akova M. Alastruey-Izquierdo A. Arikan-Akdagli S.. (2024). Invasive Fungal Diseases in Adult Patients in Intensive Care Unit (FUNDICU): 2024 consensus definitions from ESGCIP, EFISG, ESICM, ECMM, MSGERC, ISAC, and ISHAM. Intensive Care Med. 50, 502–515. doi: 10.1007/s00134-024-07341-7 38512399 PMC11018656

[B4] Blot S. I. Taccone F. S. Van den Abeele A. M. Bulpa P. Meersseman W. Brusselaers N.. (2012). A clinical algorithm to diagnose invasive pulmonary aspergillosis in critically ill patients. Am. J. Respir. Crit. Care Med. 186, 56–64. doi: 10.1164/rccm.201111-1978OC 22517788

[B5] Contou D. Dorison M. Rosman J. Schlemmer F. Gibelin A. Foulet F.. (2016). Aspergillus-positive lower respiratory tract samples in patients with the acute respiratory distress syndrome: a 10-year retrospective study. Ann. Intensive Care 6, 52. doi: 10.1186/s13613-016-0156-2 27294891 PMC4906097

[B6] Coulon P. Cordier C. Saint-Leger P. Lambiotte F. Loridant S. Mazars E. (2020). Invasive pulmonary aspergillosis in an ICU patient with Legionnaires' disease: A diagnostic challenge. J. Mycol Med. 30, 100985. doi: 10.1016/j.mycmed.2020.100985 32418638

[B7] Denning D. W. (2024). Global incidence and mortality of severe fungal disease. Lancet Infect. Dis. 24, e428–e438. doi: 10.1016/S1473-3099(23)00692-8 38224705

[B8] De Pauw B. Walsh T. J. Donnelly J. P. Stevens D. A. Edwards J. E. Calandra T.. (2008). Revised definitions of invasive fungal disease from the European Organization for Research and Treatment of Cancer/Invasive Fungal Infections Cooperative Group and the National Institute of Allergy and Infectious Diseases Mycoses Study Group (EORTC/MSG) Consensus Group. Clin. Infect. Dis. 46, 1813–1821. doi: 10.1086/588660 18462102 PMC2671227

[B9] Donnelly J. P. Chen S.C. Kauffman C. A. Steinbach W. J. Baddley J. W. Verweij P. E.. (2020). Revision and update of the consensus definitions of invasive fungal disease from the european organization for research and treatment of cancer and the mycoses study group education and research consortium. Clin. Infect. Dis. 71, 1367–1376. doi: 10.1093/cid/ciz1008 31802125 PMC7486838

[B10] Hamam J. Navellou J. C. Bellanger A. P. Bretagne S. Winiszewski H. Scherer E.. (2021). New clinical algorithm including fungal biomarkers to better diagnose probable invasive pulmonary aspergillosis in ICU. Ann. Intensive Care 11, 41. doi: 10.1186/s13613-021-00827-3 33683480 PMC7938267

[B11] Hatzl S. Scholz L. Posch F. Eller P. Reisinger A.C. Zacharias M.. (2024). Invasive pulmonary aspergillosis in critically ill patients with hantavirus infection, Austria. Emerg. Infect. Dis. 30, 1275–1278. doi: 10.3201/eid3006.231720 38782377 PMC11138969

[B12] Heldt S. Prattesb J. Eigla S. Spiessd B. Flicka H. Rabensteiner J.. (2018). Diagnosis of invasive aspergillosis in hematological Malignancy patients: Performance of cytokines, Asp LFD, and Aspergillus PCR in same day blood and bronchoalveolar lavage samples. J. Infect. 77, 235–241. doi: 10.1016/j.jinf.2018.05.001 29972764 PMC6097945

[B13] Inoue K. Muramatsu K. Nishimura T. Fujino Y. Matsuda S. Fushimi K.. (2022). Association between early diagnosis of and inpatient mortality from invasive pulmonary aspergillosis among patients without immunocompromised host factors: a nationwide observational study. Int. J. Infect. Dis. 122, 279–284. doi: 10.1016/j.ijid.2022.05.048 35643307

[B14] Ku Y. H. Chan K. S. Yang C. C. Tan C. K. Chuang Y. C. Yu W. L. (2017). Higher mortality of severe influenza patients with probable aspergillosis than those with and without other coinfections. J. Formos Med. Assoc. 116, 660–670. doi: 10.1016/j.jfma.2017.06.002 28647219

[B15] Kuo C. W. Wang S. Y. Tsai H. P. Su P. L. Cia C. T. Lai C. H.. (2022). Invasive pulmonary aspergillosis is associated with cytomegalovirus viremia in critically ill patients - A retrospective cohort study. J. Microbiol. Immunol. Infect. 55, 291–299. doi: 10.1016/j.jmii.2021.03.005 33840605

[B16] Lin C. Y. Liu W. L. Chang C. C. Chang H. T. Hu H. C. Kao K. C.. (2017). Invasive fungal tracheobronchitis in mechanically ventilated critically ill patients: underlying conditions, diagnosis, and outcomes. Ann. Intensive Care 7, 9. doi: 10.1186/s13613-016-0230-9 28083768 PMC5233606

[B17] Lin C. Y. Huang H. Y. Hsieh M. H. Fang Y. F. Lo Y. L. Lin S. M.. (2022). Impacts of nontuberculous mycobacteria isolates in non-cystic fibrosis bronchiectasis: A 16-year cohort study in Taiwan. Front. Microbiol. 13, 868435. doi: 10.3389/fmicb.2022.868435 35509319 PMC9058169

[B18] Meersseman W. Vandecasteele S. J. Wilmer A. Verbeken E. Peetermans W. E. Van Wijngaerden E. (2004). Invasive aspergillosis in critically ill patients without Malignancy. Am. J. Respir. Crit. Care Med. 170, 621–625. doi: 10.1164/rccm.200401-093OC 15229094

[B19] Mercier T. Castagnola E. Marr K. A. Wheat L. J. Verweij P. E. Maertens J. A. (2021). Defining galactomannan positivity in the updated EORTC/MSGERC consensus definitions of invasive fungal diseases. Clin. Infect. Dis. 72, S89–S94. doi: 10.1093/cid/ciaa1786 33709125

[B20] Nyga R. Maizel J. Nseir S. Chouaki T. Milic I. Roger P. A.. (2020). Invasive tracheobronchial aspergillosis in critically ill patients with severe influenza. A Clin. Trial. Am. J. Respir. Crit. Care Med. 202, 708–716. doi: 10.1164/rccm.201910-1931OC 32407157

[B21] Pardo E. Lemiale V. Mokart D. Stoclin A. Moreau A. S. Kerhuel L.. (2019). Invasive pulmonary aspergillosis in critically ill patients with hematological Malignancies. Intensive Care Med. 45, 1732–1741. doi: 10.1007/s00134-019-05789-6 31599334

[B22] Rath P. M. Steinmann J. (2018). Overview of commercially available PCR assays for the detection of aspergillus spp. DNA Patient Samples. Front. Microbiol. 9, 740. doi: 10.3389/fmicb.2018.00740 29740403 PMC5928133

[B23] Scharmann U. Kirchhoff L. Hain A. Buer J. Koldehoff M. Steinmann J.. (2021). Evaluation of three commercial PCR assays for the detection of azole-resistant aspergillus fumigatus from respiratory samples of immunocompromised patients. J. Fungi (Basel) 7. doi: 10.3390/jof7020132 PMC7916969 33670173

[B24] Schauwvlieghe A. Rijnders B. J. A. Philips N. Verwijs R. Vanderbeke L. Van Tienen C.. (2018). Invasive aspergillosis in patients admitted to the intensive care unit with severe influenza: a retrospective cohort study. Lancet Respir. Med. 6, 782–792. doi: 10.1016/S2213-2600(18)30274-1 30076119

[B25] Shi C. Shan Q. Xia J. Wang L. Wang L. Qiu L.. (2022). Incidence, risk factors and mortality of invasive pulmonary aspergillosis in patients with influenza: A systematic review and meta-analysis. Mycoses 65, 152–163. doi: 10.1111/myc.13410 34882852 PMC9306612

[B26] Tudesq J. J. Peyrony O. Lemiale V. Azoulay E.. (2019). Invasive pulmonary aspergillosis in nonimmunocompromised hosts. Semin. Respir. Crit. Care Med. 40, 540–547. doi: 10.1055/s-0039-1696968 31585479

[B27] Zou M. Tang L. Zhao S. Zhao Z. Chen L. Chen P.. (2012). Systematic review and meta-analysis of detecting galactomannan in bronchoalveolar lavage fluid for diagnosing invasive aspergillosis. PloS One 7, e43347. doi: 10.1371/journal.pone.0043347 22905261 PMC3419176

